# Supplementary material for: Identification of photoperiod-induced specific miRNAs in the adrenal glands of Sunite sheep (Ovis aries)
Source: Front Vet Sci. 2022 Jul 22;9:888207. doi: 10.3389/fvets.2022.888207 (PMC9354845; doi:10.3389/fvets.2022.888207)
Supplement: Supplementary file 1 [file Data_Sheet_1.ZIP › Supplementary file/miRDeep2 file.zip!/miRDeep2 file/Know_Structure/SP42A3/oar-mir-200b.pdf]

miRBase precursor	: oar-mir-200b
Total read count	: 193
oar-miR-200b read count	: 190
remaining reads	: 3

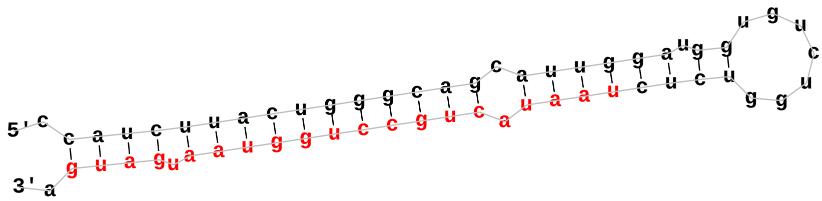

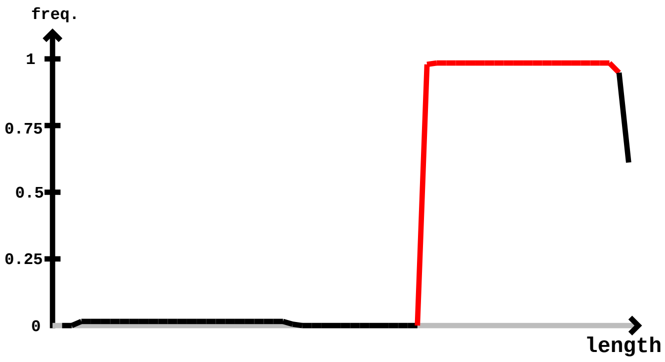


```
                                    oar-miR-200b
5'- ccaucuuacugggcagcauuggauggugucuggucucuaauacugccugguaaugaugauga   -3'    exp
    .(((((((((((((((.((((((.((.......)))))))).))))))))))).)))).    reads      mm         sample
    .caucuuacugggcagcauugga....................................    2          0          seq
    .caucuuacugggcagcauuggau...................................    1          0          seq
    .....................................uaauacugccugguaaugau..    7          0          seq
    .....................................uaauacugccugguaaugaug.    64         0          seq
    .....................................uaauacugccugguaaugauga    118        0          seq
    ......................................aauacugccugguaaugaug.    1          0          seq
```
